# Duke Research at Pickett: The evolution of a free-standing research site partnering with communities toward health equity advancement

**DOI:** 10.1017/cts.2024.649

**Published:** 2024-10-28

**Authors:** Laura Van Althuis, Sally Taylor, Debra Freeman, Stephanie Freel, Lynn Sutton, Kenisha Bethea, Leatrice Martin, Amanda McMillan, Diane Williams Garber, Keisha Bentley-Edwards, Nadine Barrett, Denise C. Snyder, Susanna Naggie

**Affiliations:** 1 Duke University School of Medicine, Duke Office of Clinical Research, Durham, NC, USA; 2 Duke University School of Medicine, Duke Clinical & Translational Science Institute, Durham, NC, USA; 3 Wake Forest University School of Medicine, Social Science and Health Policy, Division of Public Health Sciences, Winston-Salem, NC, USA

**Keywords:** community engagement, research infrastructure, CTSA, diversity, research training, education

## Abstract

While clinical research intends to improve health outcomes for all, access to research participation is often limited and inequitable. Geographic proximity is a recognized barrier, thus, systemic infrastructure solutions through federal programs including General Clinical Research Centers and Clinical and Translational Science Awards have sought to improve accessibility. Even with such support, academic medical centers often have limited clinical research-dedicated space apart from shared exam rooms in difficult-to-navigate hospitals or clinics. In 2019, the Duke University School of Medicine looked beyond its medical center campus to identify free-standing sites within Durham communities for participant study visits. Catalyzed by the COVID-19 pandemic, Duke Research at Pickett, a 22 000-square-foot building with a laboratory, 30 exam rooms, and on-site parking, opened in October 2020 to support vaccine and treatment trials. Upon the lifting of many COVID-19 restrictions, and in partnership with the Research Equity and Diversity Initiative (READI) Community Advisory Council, the building was transformed to encourage community gatherings, education, and training programs. To date, Duke Research at Pickett has hosted 2692 participants in 78 research trials and 14 community-engaged activities.

## Introduction

Ensuring equitable access to research is critical to make certain that new medical discoveries are translatable to all people. However, this can be affected by many factors including the convenience and accessibility of locations, community trust in research and associated medical providers, and a myriad of other social factors [1,2]. National Institutes of Health (NIH)-funded systematic approaches for providing dedicated research spaces have included the longstanding General Clinical Research Center Program, and an expansion in 2006 to focus on additional opportunities to improve accessibility via the Clinical and Translational Science Awards (CTSA) Program. In 2012, the transformative vision of the Institute of Medicine (IOM) committee placed heavy emphasis on community engagement throughout the research continuum, beginning with active and substantial community participation. Among other needs to facilitate community engagement, the IOM cited infrastructure development in universities and communities as one key input to successfully meet the long-term goal of improving population health. This represents an evolutionary integration of support for physicians, researchers, and communities working together to move health research from bench to community with the underlying goal of increasing access to participation in clinical research [3]. However, with this notable attention and available funding, accessibility to research for many individuals remains a challenge.

Barriers to participation in research are multifactorial and span from sociological to logistical. Most research is conducted within academic medical centers that often require paying for parking, navigating complex healthcare systems, and waiting in clinical spaces that may not feel safe or comfortable for many community members. Underserved racial and ethnic groups are often underrepresented in clinical research due to lack of invitation or exclusion from research, mistrust of research and healthcare institutions, and social factors [4]. Logistical and financial barriers to research participation, including significant time commitments, and costs relating to transportation, missed work, and child care, all impede attempts to improve inclusivity in the research populations [1]. Geographic limitations also limit access for rural communities whose members have to travel great distances to access clinical research [5].

Diversity among research participants continues to lag behind community demographics, resulting in a lack of applicability for many community members. The homogeneity of research participants, whether by age, gender, race, ethnicity, or socioeconomic status, prevents the generalizability of findings and masks potential confounders. Partnered co-design of studies that align with the health priorities of communities most likely to benefit from study results remains the exception, not the norm. Models of bi-directional engagement between participants and researchers to inform study development and implementation in its earliest stages have proven successful, producing high levels of satisfaction amongst community members and investigators alike [6]. Common successful approaches for recruiting underserved racial and ethnic groups include involving community advisory boards to align study designs with community health priorities, collaborating with community-based organizations, and engaging in community-based locations [7]. This suggests that building organizational and physical infrastructure to support community collaboration and foster trust-building relationships with a diverse group of community members can positively impact the engagement of diverse communities in research.

Moreover, logistical and geographical barriers to participating in clinical research represent uniquely actionable factors. Duke has undertaken a multifactorial initiative called the Research Equity and Diversity Initiative (READI), led by a Community Advisory Council (CAC) with representation from diverse community-based organizations. READI is organized into working groups focused on the prioritization of community partnership in research, research workforce diversity, improved research infrastructure to support inclusion for research participation, and addressing trust and trustworthiness of academic medicine through evaluation and solution finding. Here we describe one initiative within the larger READI to create a free-standing clinic dedicated to accessible research participation, educational and training programs, and community engagement.

## Establishing a free-standing research clinic amid a pandemic

Duke Health, comprising the Duke University Health System and the Duke University Schools of Medicine (SOM) and Nursing, has a large site-based clinical research program in Durham, North Carolina. There are 24 clinical research units that are the operating business units responsible for clinical trials and research studies. These units enrolled more than 29 400 participants in 2000 studies in the fiscal year 2024 and are primarily supported by a diverse portfolio of industry, state, foundation, and federal funding. While many studies are conducted in inpatient settings, others interact with research participants in central and outlying clinics, and occasionally in university-owned or leased administrative buildings.

To improve participant and researcher experiences for intensive phase I research studies, the SOM created a research-only space in the heart of its medical center dedicated to early-phase clinical trials. The Duke Early Phase Research Unit (DEPRU), established in 2008, includes a state-of-the-art clinical research confinement unit, dedicated 24/7 medical staff coverage, and a processing laboratory. This unit offers an effective model for a research-dedicated site. However, both its central location within the medical center complex and the higher costs required for managing high-risk early-phase clinical trials make the space less desirable for lower-risk studies and community research collaborations.

In the fall of 2019, following feedback from investigators across Duke, the SOM evaluated the need for off-site research space. At the conclusion of these discussions, several needs were identified: a research-dedicated outpatient space; a centralized, well-outfitted laboratory; investigational product infusion capability; a 65-foot by 5-foot area for mobility testing free of obstructions; secured cabinets and closets for equipment and study document storage; and offices for research staff. The SOM began searching for a facility to fulfill these needs with the key evaluation criteria of (1) proximity to the Duke University Medical Center campus, (2) accessibility via public transportation and free on-site parking for participants, and (3) ease and affordability of remodeling to accommodate stated needs.

In March 2020, only a few months after completing the needs assessment, the COVID-19 pandemic created an urgency in the search for a research-dedicated space. As SARS-CoV-2 research needs rapidly expanded, clinical studies involving hospitalized patients with COVID-19 and studies for vaccinated participants opened. Safe spaces for follow-up study visits after discharge or post-exposure were urgently needed. Additionally, outpatient research areas large enough to accommodate healthy participants for COVID-19 vaccine trials were lacking. Neither the health system nor current research infrastructure, such as DEPRU, could accommodate such needs while continuing to serve vulnerable patients and participants. An opportunity manifested in an available space for lease previously used as an outpatient medical clinic in the Durham community. This two-story, 22 000-square-foot building offered 30 exam rooms with sinks, a laboratory space, plenty of on-site ground-level parking, and immediate access to public transportation. Clinic renovations began in August 2020 to update information technology for compatibility with health system requirements. Duke Research at Pickett (R@P) officially opened on October 5, 2020, only 3 months after identifying the building. Vaccine studies began on premise one week later and investigational monoclonal antibody IV-infusion treatment studies began on-site within a month.

A core research staffing team was developed to support the clinic’s research operations. As many non-essential studies were paused early in 2020, staff willing and able to work with participants with COVID-19 were hired to form a central research support team working from R@P and serving COVID-19 studies in hospitals, clinics, homes, and community centers. An intense hiring focus was placed on bilingual clinical research coordinators to support the large Latine communities in Durham and the surrounding areas that were significantly impacted by COVID-19. Together with the R@P facility, the staffing team became a validated research service center (Core) in January 2021, serving investigators and studies across the research enterprise.

**Figure 1.**
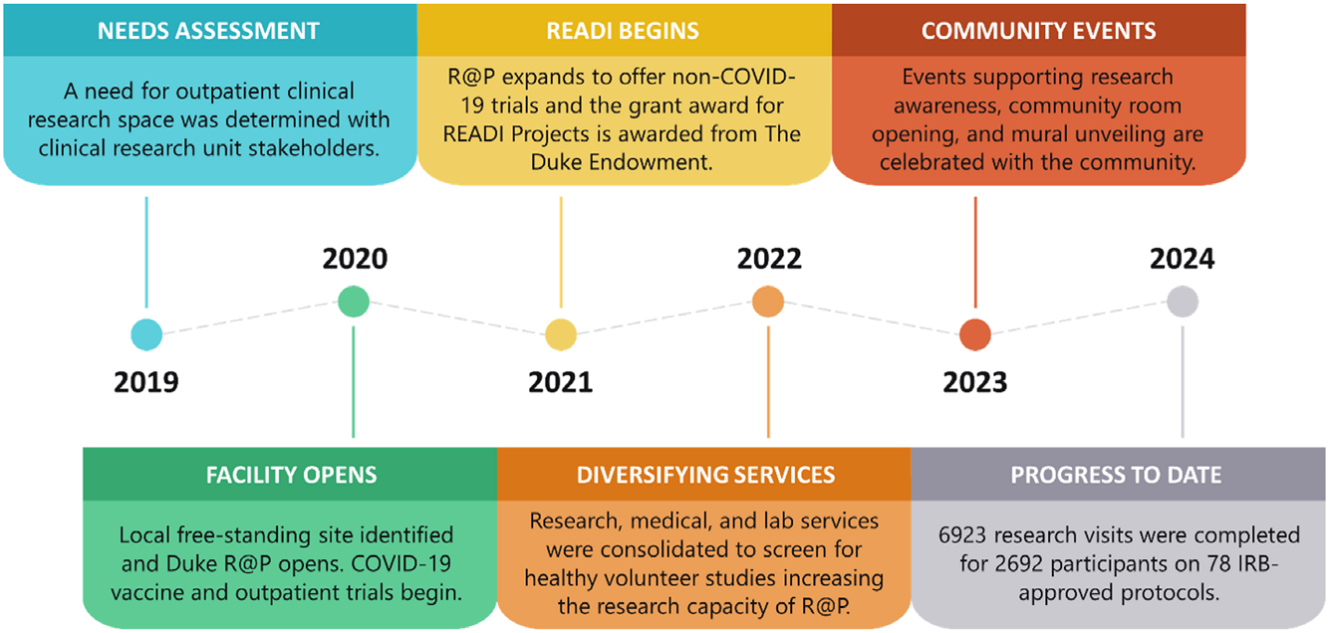
Duke Research at Pickett milestones by year.

In July 2021, R@P expanded operations to accommodate research across all disease states and populations, including pediatrics and geriatrics, using four clinic zones to safely separate participant groups, including a zone that remained dedicated to COVID-19 studies and a zone dedicated to pediatric participants. Between 2021 and 2022, the service center broadened its services to support the gradual transition of screening visits for healthy volunteer studies and lower-risk study visits from DEPRU to the more accessible R@P. Meanwhile, a multicultural team of clinical research staff continued to expand to ultimately include >30 research, regulatory, and nurse coordinators, typically 25% of whom are bilingual in Spanish and English. To date, an overall portfolio of 78 studies across 13 clinical research units have been served at the R@P site (Table 1). A timeline depicting the milestones of this project is shown in Figure 1.

**Table 1.** Use of Pickett Road Clinic from January 2021 to March 2024 by clinical research units

	n	%
Research Protocols	78	
Clinical Research Unit		
Medicine	17	21.8%
Pediatrics	15	19.2%
DEPRU	10	12.8%
DHVI	9	11.5%
Anesthesiology	8	10.3%
Surgery	5	6.4%
Emergency Medicine	4	5.1%
CTSI	3	3.8%
Heart Center	2	2.6%
Oncology	2	2.6%
Population Health Sciences	1	1.3%
School of Nursing	1	1.3%
Orthopedic Surgery	1	1.3%

DEPRU = Duke Early Phase Research Unit; DHVI = Duke Human Vaccine Institute; CTSI = Clinical and Translational Science Institute.

## Community engagement in research

While the NIH impetus to support community-engaged research has continued to strengthen, our READI CAC members noted that neighborhood communal and clinical space dedicated to authentic health and research engagement was lacking. As the safety isolation needs of the COVID-19 pandemic decreased and with this community feedback in mind, we recognized a unique opportunity to reimagine R@P as an accessible site for community use and academic partnership in research and health-focused projects. This new vision included R@P as a neighborhood-embedded outpatient clinical research site from which to base community-engaged initiatives while continuing to serve the Durham community, Duke patients, and researchers. Among READI goals, restructuring R@P facilities and services best utilized to maximize community engagement outreach activities that promote awareness, education, and participation in clinical research among underserved and marginalized populations and functioning as a training site for equity-focused workforce development programs. The READI CAC was a fully engaged partner in this work, each bringing together their lived experiences, expertise, and unique perspectives to inform and provide recommendations to guide the strategies and priorities for R@P use. The CAC comprises 19 members representing 15 community-based organizations including, patients, faith leaders, community-based clinic providers, community-based healthcare organizations, and county service representatives.

## Community-engaged research voucher program

In partnership with READI, R@P has served as the primary location for a community-engaged research voucher program, developed to fund investigator-initiated clinical research projects that incorporate community engagement principles including alignment with community health priorities and partnerships with community organizations. The research foci and engagement requirements for the voucher program were determined by the CAC and READI leadership together using a nominal group technique facilitated by project managers within the Duke Community Engaged Research Initiative team. The project managers used the 2020 Durham County Community Health Assessment priorities, identified by local community health organizations, as conversation starters with the CAC to determine priority areas for voucher-supported research. Voucher requirements included in brief: a research focus on a health disparity that disproportionately affects underserved or minoritized individuals, projects that include community members as partners, projects that include a compelling plan for recruitment of individuals from the impacted communities, and projects that could be enhanced by the voucher support provided. READI voucher support included the use of the R@P facility and/or clinical research professional (CRP) staffing support for participant recruitment, and statistical and data management, all managed through the Duke Office of Clinical Research Service Center (Core). Awards were made following a formal request for applications. A review process by Duke faculty and READI CAC members ranked top candidates. Three awards totaling $195,000 to support five clinical research studies focusing on health conditions that disproportionately affect underserved populations in the Durham community were selected for funding having met the requirements set for the voucher program.

## Creating a culturally reflective research site

**Figure 2.**
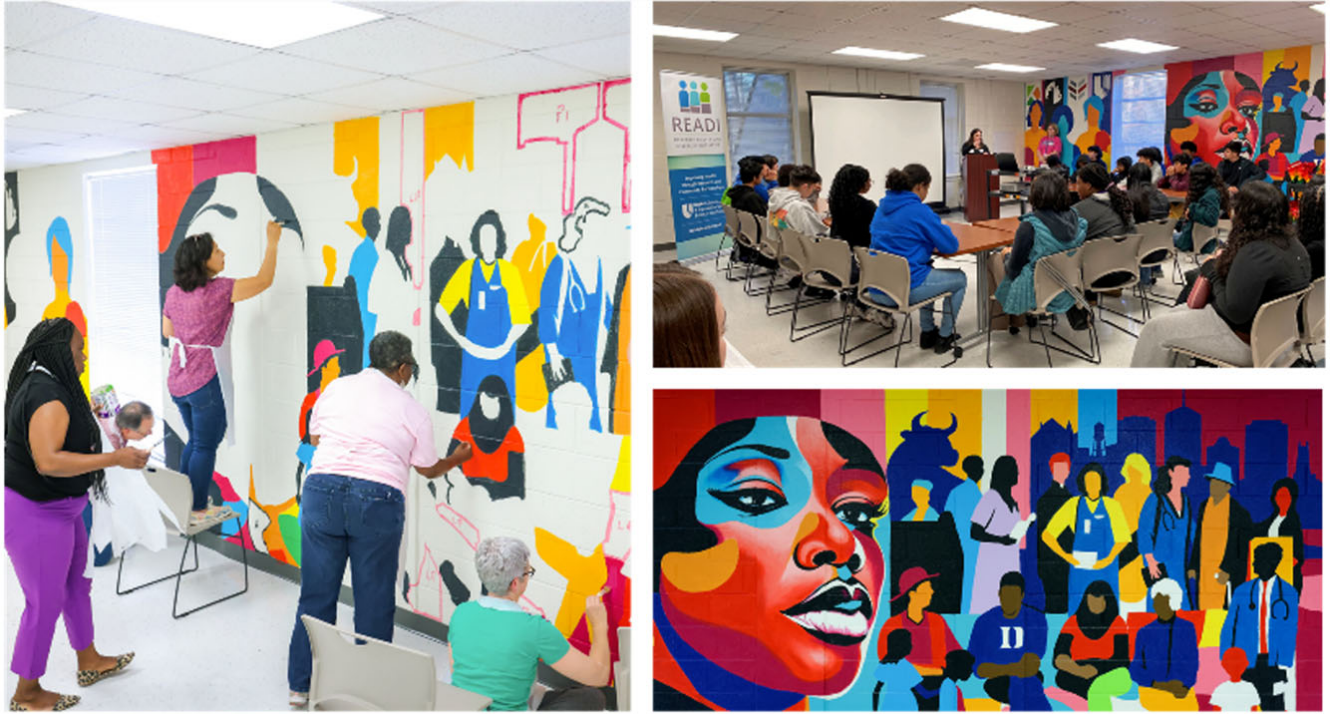
Mural painting and engaging local students. **Left**: Research faculty and staff paint the mural in the Research Equity and Diversity Initiative (READI) Community Room. **Top right:** Local high school students are engaged to learn about clinical research professional roles supporting research. **Bottom right:** The 34-foot-wide mural designed by Max Dowdle symbolizes the mission of READI while depicting the vibrant nature of the greater Durham community.

Following recommendations from the READI CAC, multiple improvements were made to the R@P facility to create welcoming spaces for community use, engagement, and outreach. In 2023, a large, minimally used training room was transformed into a vibrant community meeting room, which is available at no cost to community organizations engaging in health awareness and research activities. The community room includes audio-visual equipment and mobile tables and chairs to provide flexible seating arrangements. A mural depicting the greater Durham community and expressing visual concepts of equity, diversity, and community-driven health was commissioned to adorn its walls (Figure 2). Volunteers from a variety of Durham communities were involved throughout the artistic process from the selection of the artist and creation of the mural design to the painting and installation of the mural. An event to unveil the mural brought together over 200 residents from the Durham community and surrounding areas, including researchers, local community-based organizations, youth, and others [8].

In the following 6 months, the space was used for community discussions of clinical research ethics, a celebration and recognition of students completing the Duke CTSA/Durham Technical Community College Clinical Research Equity Scholars program [9], and a career pathway program for high school and undergraduate students focused on clinical research as a profession. Outdoor spaces have been furnished with tables and benches for use by visitors and research staff overlooking a pollinator garden and a Little Free Library installed for two Eagle projects by gender and neuro-inclusive scout troops. Information about the space is continually disseminated to local partners and at community events with the goal of increasing usage of the space and, by extension, spreading awareness of the benefits of clinical research engagement. Since renovation of R@P, the site has become a regular meeting place for Together for Resilient Youth, a coalition centered on health, wellness, and safety for all in the Durham community, community advisory boards such as for the American Society of Hematology, patient advocate consent procedure training, and multiple family support programs for those suffering from dementia.

## Multiuse for research training/education

The advent of a well-configured, multipurpose room within a designated research clinic space has afforded unique opportunities for training the current research workforce and engaging the next generation of CRPs. Even before the official launch of the READI community room, R@P was recognized as the ideal location for centrally provided education for CRPs and local students alike. Phlebotomy training, a required hands-on experience for all non-licensed CRPs who draw blood for research protocols, is available in this expansive space with room for several phlebotomy chairs and ready access to other materials and supplies. Large meetings and an institution-wide revalidation of Clinical Research Nurse Coordinators also leverage the facility. In addition to internal workforce training and education, R@P is ideal for interns and student learners, due to easy access, the variety of therapeutic areas and study types, and the large team of clinical research professionals. Local community colleges and Historically Black Colleges and Universities (HBCUs) have academic agreements with Duke to serve as an internship site for students in CRP programs. For the past 2 years, interns have had heightened exposure to a wide variety of research tasks across diverse therapeutic areas while interacting with the numerous CRP mentors who staff the research site. Moreover, because R@P and DEPRU are synergistic sites under the Duke Office of Clinical Research umbrella, interns also learn about research coordination and experience study visit activities that are unique to early-phase research, covering the entire translational spectrum from phase 1 to phase 4 clinical research and trials.

Importantly, the focus on ensuring ease of access that factored into making R@P a welcoming site for community members has likewise created an ideal space for engaging young learners in clinical research. Collaboration with the Duke Building Opportunities and Overtures in Science and Technology program has brought middle school students into the clinic to interact with CRPs, learn about studies, and role-play clinical research activities in real research spaces. Through a new partnership with Durham Public Schools and the Durham Technical Community College, work-based learning experiences including summer camps, symposia, CRP shadowing, and internships for high school students will be created. This marks an expansion of Duke’s K-12 science, technology, engineering, and mathematics programming to include clinical research and provides early-career pathways from high school into clinical research professions.

## Limitations

Despite having addressed a great need within the institution and community, limitations to the current free-standing research site remain. Geographically located in a suburban neighborhood, R@P is separated from the highest concentrations of community populations in downtown Durham that have been traditionally underserved in health care and research. As READI progresses, there will need to be consideration in how we address this issue. There is interest in developing additional sites for community engagement and identifying sites that are viewed by the community as “in the community.” The R@P site was chosen because it met the overall needs for research and community engagement but is not the optimal location. For example, the ideal location would be accessible via personal automobile and public transportation. R@P has free surface lot parking immediately adjacent to the building. However, although the site had previously been on a public transportation route, after signing the lease, it was discovered that the bus stop had been moved, leaving the closest access 0.5 miles away on a narrow-shouldered road without sidewalks. If there is sufficient need, there is the potential for a micro-shuttle to transport people from the bus stop to the R@P location, but the clinic has not yet met that threshold for Durham to invest the resources. In an effort to overcome this challenge, patient transportation has been arranged on an as-needed basis using shared ride services, paid for by the studies.

In our summative evaluation, it was clear that we need to address remaining gaps by expanding our network of community-based locations for research, encouraging and supporting more community-driven research, identifying and removing administrative barriers to community-led research, and inviting community members more frequently to fill leadership roles within our research infrastructure.

## Conclusions and future directions

Physical infrastructure is essential to supporting the needs of clinical research in a community setting, yet is often overlooked and lacks supportive funding. Clinical research sites located on large academic medical center campuses can be challenging for participants to access and navigate. Clinic schedules are often inflexible to support research visits outside normal business hours. Infrastructure development to support community-engaged research is just one piece of the solution to equity in research. Fiscal and administrative complexities at the institutional level can be difficult for community organizations to navigate, producing partnership inequities in pre- and post-award periods [10]. Moreover, diversifying the patient-facing CRP workforce is an important part of reaffirming trust in diverse communities [11].

Today, the free-standing R@P clinical research site has demonstrated its utility not only for supporting the Duke clinical research enterprise but more importantly for supporting research innovation holistically with community input and engagement. R@P has better-accommodated participants and community leaders, while community organizations have used this accessible space as a gathering place to collaborate on health, health equity, and research projects alongside staff, faculty, and students from a local HBCU and community college. Local students are engaged in on-site research-based training and education programs to explore potential career opportunities, thereby taking advantage of the experienced and multicultural team of research staff at the site. Sustaining the partnership with the READI CAC as trusted leaders for directing authentic community engagement will support the continued efforts of the facility as an important conduit for communities to embark on projects to improve health equity, dissemination, and translation.

This inclusive, multipurpose facility will continue to serve the research and health interests of the broader community while training current research staff and the next generation of CRPs. This initiative demonstrates progress toward long-term goals of increasing the diversity of research participants, fostering community trust in health and research initiatives, and translating research findings into action among underserved, under-engaged, and underrepresented communities.
